# Association of Circulating Irisin with Insulin Resistance and Metabolic Risk Markers in Prediabetic and Newly Diagnosed Type 2 Diabetes Patients

**DOI:** 10.3390/ijms27020787

**Published:** 2026-01-13

**Authors:** Daniela Denisa Mitroi Sakizlian, Lidia Boldeanu, Diana Clenciu, Adina Mitrea, Ionela Mihaela Vladu, Alina Elena Ciobanu Plasiciuc, Mohamed-Zakaria Assani, Daniela Ciobanu

**Affiliations:** 1 Doctoral School, University of Medicine and Pharmacy of Craiova, 200349 Craiova, Romania; mitroidenisa96@gmail.com; 2 Department of Microbiology, Faculty of Medicine, University of Medicine and Pharmacy of Craiova, 200349 Craiova, Romania; lidia.boldeanu@umfcv.ro; 3 Department of Diabetes, Nutrition and Metabolic Diseases, Faculty of Medicine, University of Medicine and Pharmacy of Craiova, 200349 Craiova, Romania; dianaclenciu@yahoo.com (D.C.); ada_mitrea@yahoo.com (A.M.); ionela.vladu@umfcv.ro (I.M.V.); 4 Department of Health and Motricity, Faculty of Medical and Behavioral Sciences, “Constantin Brâncuși” University of Târgu-Jiu, 210185 Târgu-Jiu, Romania; 5 Department of Immunology, Faculty of Medicine, University of Medicine and Pharmacy of Craiova, 200349 Craiova, Romania; 6 Department of Internal Medicine, University of Medicine and Pharmacy of Craiova, 200349 Craiova, Romania; elada192@yahoo.com

**Keywords:** irisin, prediabetes, type 2 diabetes mellitus, insulin resistance, hypertriglyceridemic-waist phenotype (HTGW), HOMA-IR, lipid accumulation product (LAP), atherogenic index of plasma (AIP)

## Abstract

Circulating irisin, a myokine implicated in energy expenditure and adipose tissue regulation, has been increasingly studied as a potential biomarker of metabolic dysfunction. This study evaluated the relationship between serum irisin and metabolic indices, including the atherogenic index of plasma (AIP), the lipid accumulation product (LAP), and hypertriglyceridemic-waist (HTGW) phenotype in individuals with prediabetes (PreDM) and newly diagnosed type 2 diabetes mellitus (T2DM). A total of 138 participants (48 PreDM, 90 T2DM) were assessed for anthropometric, glycemic, and lipid parameters. Serum irisin levels were measured by enzyme-linked immunosorbent assay (ELISA) and correlated with insulin resistance indices (Homeostatic Model Assessment of Insulin Resistance (HOMA-IR), Quantitative Insulin Sensitivity Check Index (QUICKI)), glycemic control (glycosylated hemoglobin A1c (HbA1c)), and composite lipid markers (total triglycerides-to-high-density lipoprotein cholesterol (TG/HDL-C)). Group differences were evaluated using non-parametric tests; two-way ANOVA assessed interactions between phenotypes and markers; multiple linear regression (MLR) and logistic regression models explored independent associations with metabolic indices and HTGW; receiver operating characteristic (ROC) analyses compared global and stratified model performance. Serum irisin was significantly lower in T2DM than in PreDM (median 140.4 vs. 230.7 ng/mL, *p* < 0.0001). Irisin levels remained comparable between males and females in both groups. Post hoc analysis shows that lipid indices and irisin primarily distinguish HTGW phenotypes, especially in T2DM. In both groups, irisin correlated inversely with HOMA-IR, AIP, and TG/HDL-C, and positively with QUICKI, indicating a possible compensatory role in early insulin resistance. MLR analyses revealed no independent relationship between irisin and either AIP or LAP in PreDM, while in T2DM, waist circumference remained the strongest negative predictor of irisin. Logistic regression identified age, male sex, and HbA1c as independent predictors of the HTGW phenotype, while irisin contributed modestly to overall model discrimination. ROC curves demonstrated good discriminative performance (AUC = 0.806 for global; 0.794 for PreDM; 0.813 for T2DM), suggesting comparable predictive accuracy across glycemic stages. In conclusion, irisin levels decline from prediabetes to overt diabetes and are inversely linked to lipid accumulation and insulin resistance but do not independently predict the HTGW phenotype. These findings support irisin’s role as an integrative indicator of metabolic stress rather than a stand-alone biomarker. Incorporating irisin into multi-parameter metabolic panels may enhance early detection of cardiometabolic risk in dysglycemic populations.

## 1. Introduction

Type 2 diabetes mellitus (T2DM) is one of the most significant global health challenges, with a steadily increasing prevalence and a significant contribution to morbidity and mortality through cardiovascular and metabolic complications [1,2]. Prediabetes (PreDM), defined by intermediate fasting plasma glucose levels and/or impaired glucose tolerance, represents a high-risk state for the development of T2DM and is already associated with metabolic and vascular alterations [3,4,5,6]. The identification of circulating biomarkers that reflect the transition from PreDM to overt T2DM is essential for early diagnosis, risk stratification, and the development of personalized interventions [7].

Beyond traditional risk factors—obesity, insulin resistance, dyslipidemia, and low-grade chronic inflammation—recent research has highlighted the role of myokines, bioactive peptides secreted by skeletal muscle that act as endocrine and paracrine mediators [8,9]. These molecules link physical activity and muscle metabolism with systemic energy balance, inflammation, and glucose homeostasis, providing a functional bridge between skeletal muscle and key metabolic organs such as the liver, adipose tissue, and pancreas [10].

Among myokines, irisin has attracted particular attention. First described in 2012, irisin is generated by cleavage of the fibronectin type III domain-containing protein 5 (FNDC5) under the control of peroxisome proliferator-activated receptor gamma coactivator 1-alpha (PGC-1α), a master regulator of energy metabolism [11]. Experimental studies have shown that irisin induces the “browning” of white adipose tissue, increases energy expenditure, and exerts pleiotropic effects on glucose and lipid metabolism [11,12]. Moreover, irisin appears to improve insulin sensitivity, attenuate systemic inflammation, and support endothelial function in both animal models and in vitro experiments [13,14].

Despite these promising findings, clinical data on circulating irisin concentrations in glucose-metabolism disorders remain inconsistent. Several studies have reported lower irisin levels in patients with T2DM compared with healthy controls [15,16,17,18]. In PreDM, the evidence is even more heterogeneous, with some studies suggesting that irisin reflects early insulin resistance and subtle metabolic dysfunctions [19,20,21,22,23,24]. Furthermore, the associations between irisin and anthropometric variables, lipid markers, and insulin resistance indices (e.g., Homeostatic Model Assessment of Insulin Resistance (HOMA-IR)) remain poorly defined [25,26].

Beyond glucose and insulin-related indices, lipid-derived composite markers such as the Atherogenic Index of Plasma (AIP) and the Lipid Accumulation Product (LAP) have gained increasing recognition as valuable predictors of metabolic and cardiovascular risk. AIP, calculated as the logarithmic ratio of triglycerides (TG) to high-density lipoprotein cholesterol (HDL-C), reflects the balance between atherogenic and protective lipoprotein particles and has been shown to correlate strongly with insulin resistance, endothelial dysfunction, and incident T2DM [27,28,29,30]. Similarly, LAP, an index combining waist circumference and fasting triglycerides, serves as a surrogate marker of visceral adiposity and lipid overaccumulation, outperforming traditional anthropometric measures in predicting progression to metabolic syndrome and diabetes [31,32]. Both AIP and LAP have demonstrated superior predictive value for cardiometabolic complications and subclinical atherosclerosis in individuals with prediabetes and T2DM, emphasizing their utility as integrative markers linking dyslipidemia and central obesity [33,34].

In addition to classical markers of metabolic dysfunction, the hypertriglyceridemic-waist (HTGW) phenotype—defined by the coexistence of elevated waist circumference (WC) and high TG levels—has emerged as a simple yet powerful clinical predictor of cardiometabolic risk and visceral adiposity [35,36]. Individuals with HTGW exhibit a clustering of insulin resistance, low-grade inflammation, and atherogenic dyslipidemia, making this phenotype a valuable surrogate for identifying patients at high risk of T2DM and cardiovascular disease [37,38,39].

Since myokines, such as irisin, are closely linked to energy expenditure, body fat, and fat metabolism [11,21,40,41], their potential association with HTGW is particularly interesting. However, limited data currently exist on how circulating irisin levels relate to specific metabolic patterns, including HTGW. Exploring this link could improve our understanding of irisin as a comprehensive biomarker, reflecting both glucose issues and lipid-related heart risk. Another challenge is the absence of standardized methods for assay measurement and validation, which hampers the ability to accurately compare results across different studies [42,43].

There is a knowledge gap regarding a consensus that suggests irisin could serve as a useful biomarker with both diagnostic and prognostic value in metabolic diseases, especially in the transition from PreDM to T2DM. Additionally, there is a lack of comparative studies directly assessing differences in circulating irisin levels between PreDM individuals and those newly diagnosed with T2DM, while also exploring correlations with insulin resistance and glycemic control. Since this transition stage is crucial for preventing long-term complications, clarifying irisin’s role could provide valuable clinical insights.

Therefore, the present study aimed to evaluate serum irisin levels and their associations with key metabolic markers, including indices of insulin resistance and lipid-related risk factors (HOMA-IR, Quantitative Insulin Sensitivity Check Index (QUICKI), TG/HDL-C, AIP, and LAP), in individuals with PreDM and newly diagnosed T2DM. Additionally, we investigated the relationships between irisin concentrations and HTGW phenotypes (normal TG and enlarged WC (NTEW), elevated TG and normal WC (ETNW), and increased TG and enlarged WC (HTGW)) to determine whether this adiposity-lipid pattern influences myokine secretion and cardiometabolic risk in early dysglycemia.

## 2. Results

### 2.1. Baseline Characteristics

Table 1 summarizes the anthropometric and metabolic characteristics of the study participants. Although participants with T2DM were slightly older than those with PreDM, the difference did not reach statistical significance (*p* = 0.155). The sex distribution was balanced across groups, whereas the proportion of urban participants was higher in the T2DM group.

Regarding body composition, subjects with T2DM had significantly higher body weight, height, WC, and hip circumference than the PreDM group (*p* = 0.003, 0.003, <0.0001, and 0.010, respectively). However, body mass index (BMI) did not differ significantly (*p* = 0.355), suggesting that body fat distribution rather than total adiposity distinguishes the two groups. Mean arterial pressure (MAP) tended to be higher in T2DM but did not reach statistical significance (*p* = 0.076).

As expected, glycemic markers showed pronounced differences: fasting plasma glucose (FPG) and glycosylated hemoglobin A1c (HbA1c) were markedly elevated in T2DM (*p* < 0.0001 for both). Fasting insulin was more than 15-fold higher in T2DM (median 49.82 µU/mL) compared with PreDM (3.28 µU/mL), resulting in significantly higher HOMA-IR and lower QUICKI indices (*p* < 0.0001), confirming severe insulin resistance in newly diagnosed diabetes.

Regarding lipid profile, TG were comparable between groups. In contrast, low-density lipoprotein cholesterol (LDL-C) was significantly higher in T2DM (*p* < 0.0001), HDL-C was lower (*p* = 0.006), and total cholesterol (TC) was slightly but significantly increased (*p* = 0.026). Derived indices, including simple atherogenic ratios (TG/HDL-C, AIP), and adiposity accumulation (LAP), were all significantly higher in T2DM (*p* < 0.0001 for all), indicating a markedly more atherogenic lipid profile and greater visceral lipid accumulation.

When categorized by HTGW phenotype, the distributions of NTEW, ETNW, and HTGW were similar between PreDM and T2DM, with approximately one-third of subjects in each group classified as HTGW.

**Figure 1:**
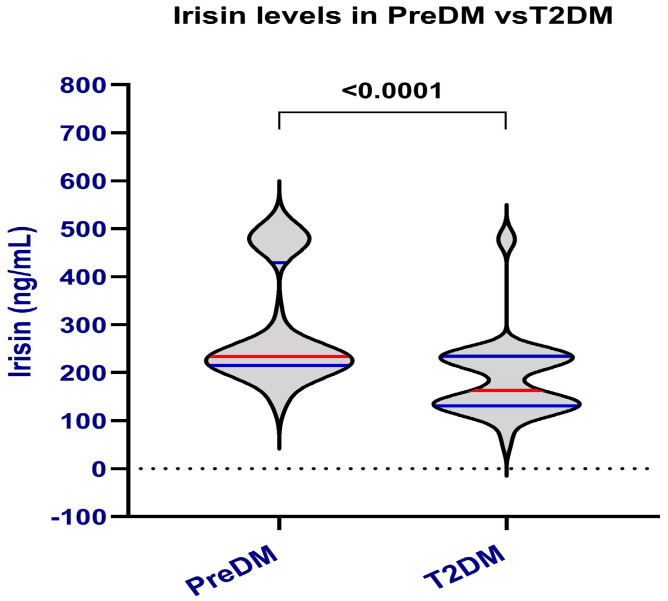
Circulating irisin levels in prediabetes (PreDM) and type 2 diabetes mellitus (T2DM). The violin plots display the distribution of Irisin in both groups. Horizontal red lines represent medians, while blue horizontal lines indicate the interquartile range (IQR). Values are shown in ng/mL. The violin plots show higher Irisin levels in PreDM subjects, with a statistically significant difference (*p* < 0.0001) according to the Mann–Whitney U test.

Serum irisin levels were significantly lower in T2DM compared with PreDM (median 140.40 vs. 230.70 ng/mL, *p* < 0.0001) (Figure 1).

### 2.2. Comparing the Irisin and Metabolic Parameters (Atherogenic Ratios and Adiposity Accumulation) Levels in the PreDM and T2DM Groups

#### 2.2.1. Comparing Irisin and Metabolic Parameters Levels According to Gender in the PreDM and T2DM Groups

Table 2 presents a comparison of irisin and metabolic parameters by gender within the PreDM and T2DM groups.

In PreDM subjects, serum irisin levels were similar between males and females (median 230.0 vs. 238.5 ng/mL, *p* = 0.915), showing no sex-related difference in circulating myokine concentration. No significant gender differences in insulin or HOMA-IR were observed (*p* > 0.05), suggesting comparable insulin sensitivity in both sexes at the prediabetic stage. Likewise, QUICKI values were similar in males and females (0.18 vs. 0.17, *p* = 0.735). Atherogenic ratios, including TG/HDL-C and AIP, were modestly higher in females (median TG/HDL-C 3.17 vs. 2.69; AIP 0.51 vs. 0.43), but these differences did not reach statistical significance (*p* = 0.077 and *p* = 0.058, respectively). In contrast, LAP was significantly higher in females than in males (67.05 vs. 40.73, *p* < 0.0001), indicating greater visceral lipid accumulation in females despite similar overall adiposity indices.

Among patients with newly diagnosed T2DM, irisin levels remained comparable between males and females (149.0 vs. 167.0 ng/mL, *p* = 0.867), confirming the absence of a gender effect on myokine concentrations in the diabetic state. Similarly, insulin, HOMA-IR, and QUICKI values did not differ significantly between sexes (*p* = 0.995, 0.554, and 0.626, respectively), suggesting equivalent degrees of insulin resistance and β-cell response in both male and female participants. For atherogenic ratios, TG/HDL-C and AIP were slightly higher in males, but the differences were not statistically significant (*p* = 0.337 and *p* = 0.307, respectively). However, LAP values were again significantly higher in females than in males (96.94 vs. 76.55, *p* = 0.004), indicating a greater visceral fat burden and lipid storage capacity in females with T2DM.

#### 2.2.2. Comparing the Irisin and Metabolic Parameters Levels According to the HTGW Phenotype in the PreDM and T2DM Groups

A two-way ANOVA (Kruskal–Wallis) test was used to evaluate the effects of the HTGW phenotype (column factor: NTEW, ETNW, HTGW) and marker type (row factor: insulin, HOMA-IR, QUICKI, TG/HDL-C, AIP, LAP, irisin) in patients with PreDM and T2DM (Table 3).

In the PreDMs group, no significant main effect of marker type was observed (*p* > 0.05), indicating that the overall levels of insulin, HOMA-IR, QUICKI, TG/HDL-C, AIP, LAP, and irisin did not differ markedly within the group. In contrast, there was a significant main effect of HTGW phenotype on atherogenic ratios including TG/HDL-C (F (2, 26) = 9.078, *p* = 0.001), AIP (F (2, 26) = 10.290, *p* = 0.001), and adiposity accumulation parameter, such us LAP (F (2, 26) = 13.330, *p* = 0.001). The interaction between insulin, HOMA-IR, QUICKI, irisin, and HTGW phenotype was not significant (*p* > 0.05), suggesting that the pattern of variation among these markers was consistent across phenotypes.

In the T2DM group, the main effect of marker type was significant (*p* < 0.0001) for insulin (F (39,48) = 2.284, *p* = 0.003), HOMA-IR (F (39,48) = 6.588, *p* < 0.0001), and QUICKI (F (39,48) = 5.452, *p* < 0.0001), showing markedly different values compared to the lipid-derived indices. A strong main effect of the HTGW phenotype was observed for TG/HDL-C (F (2,48) = 17.100), AIP (F (2,48) = 22.940), and LAP (F (2,48) = 18.580), with *p* < 0.0001 for all, confirming the metabolic impact of the HTGW pattern in T2DM. For irisin, only the main effect of HTGW phenotype (column factor) was significant (F (2,48) = 4.857, *p* = 0.012).

##### Post Hoc Analysis

As the two-way ANOVA (Kruskal–Wallis) test demonstrated significant main effects of the HTGW phenotype for several markers, post hoc pairwise comparisons were subsequently performed using the Tukey test with Holm–Bonferroni correction to identify which phenotypic groups differed significantly (Table 4).

In the PreDM group, post hoc pairwise comparisons using the Tukey test with Holm–Bonferroni correction revealed significant differences in lipid-related indices. In contrast, insulin resistance markers remained comparable across phenotypes.

TG/HDL-C, AIP, and LAP showed significantly higher values in ETNW and HTGW compared with NTEW (*p* Holm-adj ≤ 0.002 for all). In contrast, insulin, HOMA-IR, QUICKI, and irisin did not differ significantly between HTGW phenotypes (*p* Holm-adj > 0.05).

In the T2DM group, pairwise comparisons revealed greater metabolic divergence among phenotypes. TG/HDL-C, AIP, and LAP differed significantly across all comparisons, with the highest values consistently observed in the HTGW phenotype (*p* Holm-adj < 0.01 for all).

For irisin, significant differences were detected between NTEW and ETNW (*p* Holm-adj = 0.011) and between ETNW and HTGW (*p* Holm-adj = 0.018). In contrast, insulin, HOMA-IR, and QUICKI remained statistically similar among phenotypes (*p* Holm-adj > 0.05).

The post hoc analysis shows that lipid-derived indices and irisin are key discriminants among HTGW phenotypes, particularly in T2DM.

### 2.3. Regression Analyses

#### 2.3.1. Multiple Linear Regression

To further investigate whether circulating irisin remains an independent predictor of metabolic risk when evaluated alongside composite lipid indices, multiple linear regression (MLR) analyses were conducted. The models were designed to assess the independent associations of serum irisin with atherogenic and adiposity-related markers, AIP and LAP, while adjusting for glycemic control (HbA1c), age, and sex.

Two separate regression models were constructed: the first included AIP as the principal lipid-related predictor, whereas the second incorporated LAP, allowing for a differential evaluation of irisin’s relationship with plasma lipid atherogenicity versus visceral adiposity:

- *Model* 1 *(AIP)*: AIP (log10 TG/HDL) + Insulin (µU/mL) + HbA1c (%) + HOMA-IR + QUICKI + TG/HDL-C + Waist (cm) + Age (y) + Sex (Female);
- *Model* 2 *(LAP)*: LAP + Insulin (µU/mL) + HbA1c (%) + HOMA-IR + QUICKI + TG/HDL-C + Age (y) + Sex (Female).

This approach aimed to determine whether the observed associations between irisin and metabolic dysregulation persist after accounting for the overlapping effects of lipid and glycemic components.

In the PreDM group (Table 5), both MLR models—incorporating AIP or LAP as the main composite lipid indices—showed no statistically significant predictors of circulating irisin levels.

In Model 1 (AIP-based), none of the examined variables reached statistical significance (model *p* = 0.789; adj. R^2^ = −0.083). The β coefficients for AIP (β = −150.9, *p* = 0.531) and TG/HDL-C (β = +21.5, *p* = 0.617) were small and nonsignificant, suggesting no independent relationship between lipid atherogenicity and serum irisin concentrations. Likewise, insulin, HOMA-IR, and QUICKI showed no meaningful association, and multicollinearity was evident among insulin resistance markers (VIF > 20).

In Model 2 (LAP-based), results were similar (model *p* = 0.805; adj. R^2^ = −0.087), with no variable reaching statistical significance (Table 5). The β for LAP (β = 0.60, *p* = 0.627) indicated a weak and nonsignificant trend toward a positive relationship. HbA1c and age showed small inverse effects, but neither reached significance (*p* > 0.10).

Collectively, these findings indicate that in PreDM individuals, serum irisin levels are not independently determined by composite lipid indices (AIP or LAP) nor by classical insulin resistance markers. The uniformly low explanatory power of both models (adj. R^2^ ≈ 0) and high multicollinearity suggest that the associations between irisin, lipids, and insulin dynamics are complex and likely confounded by overlapping metabolic pathways rather than independent effects.

In the T2DM group (Table 6), the multiple linear regression models incorporating either AIP or LAP as the primary metabolic predictors showed trends different from those in PreDM, although none of the overall models reached statistical significance.

The model incorporating AIP, together with insulin, HbA1c, and insulin resistance indices, did not significantly predict circulating irisin levels (model *p* = 0.494, adj. R^2^ = −0.006). None of the individual variables, including AIP (β = −47.4, *p* = 0.696), insulin (β = −1.69, *p* = 0.457), or HbA1c (β = −1.07, *p* = 0.852), exhibited significant associations. However, waist circumference emerged as the only independent negative predictor (β = −1.71, *p* = 0.018), suggesting that larger central adiposity is associated with lower circulating irisin levels. This inverse relationship is consistent with the hypothesis that chronic adiposity and reduced muscle–adipose cross-talk may blunt irisin secretion in established diabetes. High collinearity between insulin-related indices (VIF > 8) indicates overlapping effects among markers of insulin resistance and lipid metabolism, potentially obscuring independent associations.

When LAP replaced AIP (Table 6) as the main lipid–adiposity marker, the model showed a slightly improved fit (model *p* = 0.261; adj. R^2^ = 0.028). LAP displayed a borderline positive association with irisin (β = 0.58, *p* = 0.092), whereas waist circumference remained a significant negative predictor (β = −3.01, *p* = 0.004). TG/HDL-C ratio showed a weak inverse trend (β = −9.68, *p* = 0.081), reinforcing the association between atherogenic dyslipidemia and lower irisin levels.

Altogether, the T2DM models suggest that central obesity and dyslipidemia jointly contribute to reduced irisin levels, while the direct influence of insulin resistance markers diminishes when adiposity indices are considered. This pattern aligns with the concept that chronic metabolic stress in T2DM primarily modulates irisin via adipose-driven mechanisms rather than glycemic control alone.

##### Parsimonious Multiple Linear Regression Models

Given the high intercorrelation among metabolic variables—particularly between indices that share standard components (e.g., HOMA-IR, insulin, and QUICKI; or AIP, TG/HDL-C, and LAP)—the initial full regression models exhibited substantial multicollinearity, limiting interpretability and inflating standard errors. To improve model stability and ensure independent contribution of each predictor, we implemented parsimonious multiple linear regression models, retaining only variables with distinct physiological relevance and low collinearity (variance inflation factor, VIF < 2). Specifically, AIP and LAP were analyzed in separate models as composite lipid-derived indices that reflect different aspects of metabolic dysfunction: AIP as a marker of plasma lipid atherogenicity, and LAP as an integrated indicator of visceral lipid accumulation. HbA1c, age, and sex were retained as covariates in all models to adjust for glycemic status and demographic influences, whereas redundant insulin resistance indices were excluded. This parsimonious approach provides a more reliable estimation of independent associations between circulating irisin and metabolic risk markers by minimizing overfitting and reducing shared variance among predictors, thereby enhancing the interpretability of the resulting β coefficients and overall model structure.

In the *PreDM* cohort (Table 7), the parsimonious model based on AIP (together with HbA1c, age, sex, and waist) explains a small proportion of the irisin variation (R^2^ = 0.104; Adj. R^2^ = −0.003), and the overall significance is insignificant (*p* = 0.444). All standardized coefficients are of small magnitude and insignificant: the effect for AIP is inverse (βstd = −0.083, *p* = 0.617), and for HbA1c, a moderate but insignificant inverse trend is observed (βstd = −0.237, *p* = 0.121). Age, sex, and waist circumference do not have relevant independent effects. These results are consistent with the FDR-adjusted correlation analysis, suggesting that irisin does not show robust independent associations with lipid atherogenicity or glycemic control when these factors are assessed simultaneously in the prediabetic stage.

In the LAP-based model, the overall fit is modest (R^2^ = 0.098, *p* = 0.335), and none of the predictors has a significant independent effect on serum irisin levels. The coefficient for LAP (βstd = −0.172, *p* = 0.288) indicates a negative association between visceral lipid accumulation and irisin, but the effect size is small. HbA1c also shows a small inverse association (βstd = −0.218, *p* = 0.144), while age and sex do not significantly influence irisin (βstd < 0.15; *p* > 0.33).

These results confirm observations from the previous model (AIP), suggesting that in PreDM, the combined lipid index LAP does not better explain irisin variation than AIP, and that the relationship between irisin, dyslipidemia, and glycemic control is weak and statistically insignificant. Thus, there is no evidence for an independent association between irisin and the composite lipid markers at this metabolic stage, which may reflect either an incipient compensatory regulatory mechanism or a significant interindividual variability in irisin secretion.

In the T2DM group (Table 8), the AIP-based model shows a modest fit (R^2^ = 0.131; Adj. R^2^ = 0.027; model *p* = 0.271), with no significant independent associations between irisin and the analyzed predictors. The standardized coefficients are of small magnitude and statistically insignificant: AIP (βstd = −0.118, *p* = 0.321) and HbA1c (βstd = −0.106, *p* = 0.330) show a weak inverse trend, without clinical or statistical relevance. Also, age and sex do not significantly influence irisin levels (*p* > 0.38), and the effect of waist circumference is a minor positive effect (βstd = 0.086; *p* = 0.433). These data confirm the absence of an independent relationship between irisin and markers of atherogenic dyslipidemia in the context of established diabetes. In contrast to the PreDM phase, in which negative trends in AIP and HbA1c were somewhat more pronounced, in T2DM, irisin variation seems less closely associated with the lipid profile, possibly reflecting a loss of irisin’s compensatory function as insulin resistance and adipocyte dysfunction worsen.

In the LAP-based model for patients with T2DM, the overall fit is modest (R^2^ = 0.154; Adj. R^2^ = 0.081), and the overall model is marginally significant (*p* = 0.087). The standardized coefficient for LAP (βstd = −0.204, *p* = 0.056) suggests a moderate inverse association between irisin and visceral lipid accumulation, approaching statistical significance. This result may reflect a clearer pathophysiological relationship between decreased irisin and the degree of visceral dyslipidemia in patients with T2DM. HbA1c and age maintain a mild inverse association (βstd = −0.112, βstd = −0.097, respectively; in both cases *p* > 0.250), and sex has no independent influence (βstd = 0.041; *p* = 0.668).

Interpreting the results of the two models (AIP and LAP) together, it is observed that LAP shows the most consistent inverse trend with irisin, although it does not reach strict statistical significance. This may suggest that the composite LAP index more faithfully reflects the relationship between visceral excess and myokinetic regulation than AIP, which predominantly describes the plasma lipid profile.

#### 2.3.2. Logistic Regression for HTGW Phenotype

##### Logistic Regression—Global Model

To further investigate whether circulating irisin is independently associated with the presence of the HTGW phenotype, a binary logistic regression was fitted within the global model, with HTGW (yes/no) as the dependent variable:

- HTGW (yes/no) = Irisin (standardized) + Age (y) + Sex (Male = 1) + BMI (kg/m^2^) + group (T2DM = 1) + HbA1c (%) + insulin (µU/mL).

Irisin values were standardized (z-scores) to express the effect per one standard deviation increase.

The global model was statistically significant and well calibrated. Irisin did not independently predict the HTGW phenotype (OR = 0.94, *p* = 0.814) after adjustment for age, sex, BMI, glycemic status, HbA1c, and insulin. Age and male sex were associated with lower odds of HTGW (OR = 0.94 per year, *p* = 0.015; OR = 0.12, *p* < 0.001), while HbA1c emerged as an independent positive predictor (OR = 1.42 per 1% increase, *p* = 0.048). BMI, insulin, and diabetes status did not show significant associations (Table 9).

**Figure 2:**
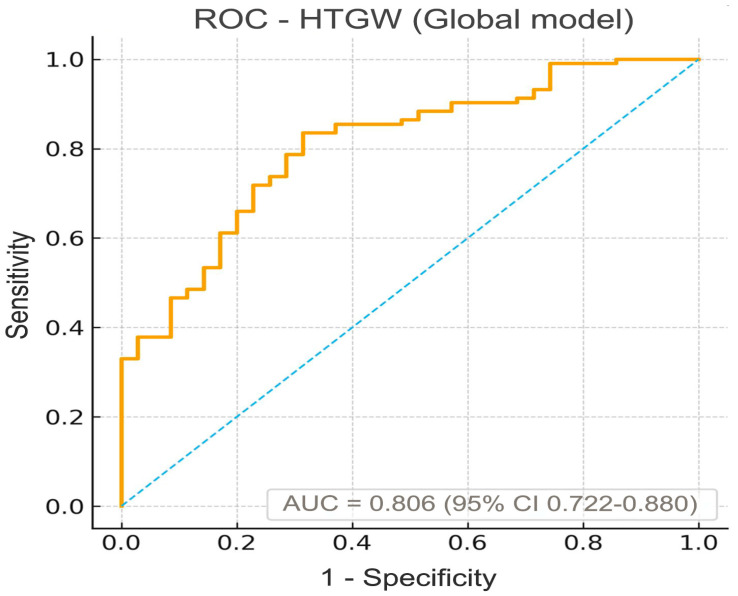
ROC curve for prediction of the hypertriglyceridemic-waist (HTGW) phenotype in the combined cohort (n = 138). The logistic model included standardized irisin (per 1 SD), age, sex, BMI, diabetes status (T2DM vs. PreDM), HbA1c, and fasting insulin. The model showed good discrimination, with an AUC of 0.806 (95% CI: 0.722–0.880; *p* < 0.001). The dashed diagonal line indicates no discrimination (AUC = 0.5); the greater the curve’s distance above this line, the better the classification performance.

The model’s AUC of 0.806 (95% CI 0.722–0.880; *p* < 0.001) (Figure 2) indicates good discriminative ability for identifying individuals with the HTGW phenotype using this covariate set, even after excluding waist circumference to avoid overadjustment.

##### Logistic Regression—Separate Multivariate Models for HTGW (Prediabetes)

In PreDM individuals (Table 10), lower circulating irisin levels were associated with higher odds of having the HTGW phenotype (OR = 0.72, *p* = 0.362), though the relationship did not reach significance. Male sex was an independent negative predictor of HTGW (*p* = 0.024), consistent with a higher prevalence of the phenotype among females. HbA1c and insulin showed weak, non-significant positive trends, while age exhibited a mild inverse association.

**Figure 3:**
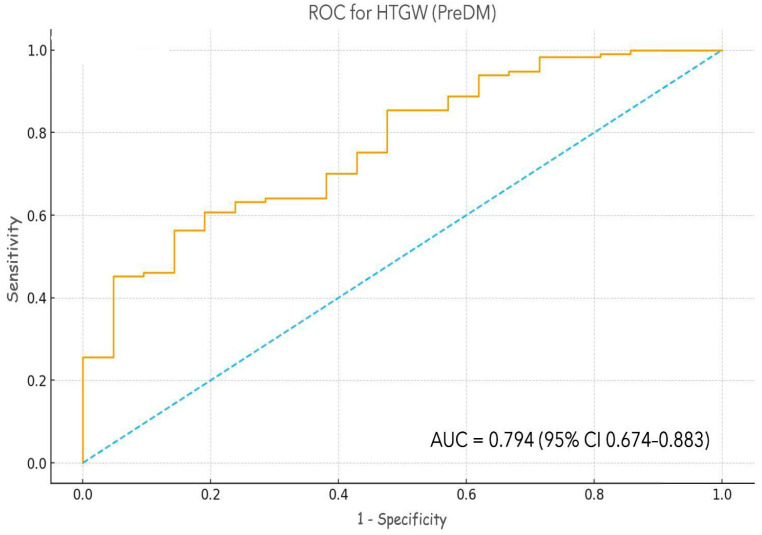
Receiver operating characteristic (ROC) curves showing the discriminative performance of multivariate logistic regression models for predicting the hypertriglyceridemic-waist (HTGW) phenotype in prediabetic (PreDM) participants. Model included standardized irisin (per 1 SD), age, sex, body mass index (BMI), glycated hemoglobin (HbA1c), and fasting insulin as independent predictors. The diagonal dashed line represents the reference line for no discrimination (AUC = 0.5). The PreDM model demonstrated an area under the curve (AUC) of 0.794 (95% CI: 0.674–0.883; *p* < 0.001), indicating fair discriminative ability.

The model had moderate explanatory power (Nagelkerke R^2^ = 0.297) and good calibration (Hosmer–Lemeshow χ^2^(8) = 6.42, *p* = 0.602), with AUC = 0.794, (*p* < 0.001) indicating fair discrimination (Figure 3).

##### Logistic Regression—Separate Multivariate Models for HTGW (T2DM)

In patients with T2DM (Table 11), irisin did not predict the HTGW phenotype independently (OR = 1.08, *p* = 0.810). However, younger age, female sex, and higher HbA1c were significantly associated with HTGW presence. HbA1c emerged as the only metabolic variable with a consistent positive association (OR = 1.45, *p* = 0.046), suggesting that poorer glycemic control contributes to the combined dyslipidemic–adiposity pattern.

**Figure 4:**
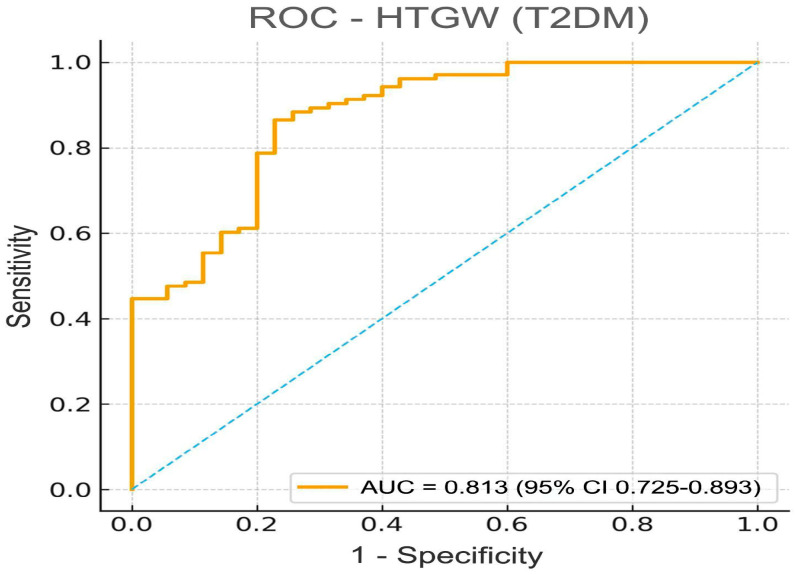
ROC curves comparing the predictive performance for the hypertriglyceridemic-waist (HTGW) phenotype in T2DM. The T2DM model achieved AUC = 0.813 (95% CI: 0.725–0.893), suggesting comparable accuracy. The model shows acceptable discrimination (*p* < 0.001), with overlapping confidence intervals, indicating no statistically significant difference between groups.

The model showed good overall fit (χ^2^(6) = 19.56, *p* = 0.003) and discrimination (AUC = 0.813, *p* < 0.001), comparable to that observed in prediabetes (Figure 4). The slight improvement in AUC among T2DM subjects likely reflects the greater contribution of hyperglycemia and insulin resistance to the combined dyslipidemic–adiposity profile.

*Comparative synthesis (PreDM* vs. *T2DM)*: Across both glycemic statuses, irisin did not independently predict the HTGW phenotype after adjusting for age, sex, BMI, HbA1c, and insulin. The direction of association was inverse in PreDM (OR < 1) and null in T2DM, supporting the hypothesis that irisin’s potential protective influence diminishes as metabolic dysfunction progresses. Both models identified female sex and higher HbA1c as consistent correlates of the HTGW phenotype, reinforcing the role of adiposity distribution and poor glycemic control as key determinants of this atherogenic metabolic profile. Discriminative performance (AUC ≈ 0.800) indicates that these models achieve good accuracy in identifying HTGW individuals across both groups.

##### ROC Analysis and Comparative Performance

The global model—including all participants irrespective of glycemic status—demonstrated excellent discriminative performance, with an AUC of 0.806 (95% CI: 0.722–0.880, *p* < 0.001). This suggests that the combination of circulating irisin, age, sex, BMI, HbA1c, and fasting insulin effectively distinguishes individuals with and without the HTGW phenotype across the metabolic spectrum.

When stratified by group, the PreDM model yielded an AUC of 0.792 (95% CI: 0.674–0.883), while the T2DM model achieved a slightly higher AUC of 0.813 (95% CI: 0.725–0.893). These values indicate comparable predictive performance between prediabetic and diabetic patients, with overlapping confidence intervals and no statistically significant difference in discrimination (*p* > 0.05).

Although both stratified models maintained high classification accuracy, the T2DM model’s marginally higher AUC likely reflects the greater contribution of glycemic dysregulation and insulin resistance to the HTGW phenotype in more advanced metabolic states. Conversely, the similar performance observed in PreDM suggests that subclinical alterations in lipid and glucose metabolism, even before the onset of overt diabetes, already contribute substantially to the emergence of this atherogenic phenotype.

Overall, the models show that the predictive value of circulating irisin remains modest and consistent across metabolic stages, while HbA1c and sex contribute more robustly to HTGW classification.

### 2.4. Correlation Analyses

Spearman correlation analyses were performed to evaluate the associations between serum irisin concentrations and a range of metabolic parameters, including insulin, HbA1c, indices of insulin resistance (HOMA-IR, QUICKI), lipid-related indices (LAP, AIP, TG/HDL-C), and waist circumference. Correlations were assessed separately for subjects with PreDM and those with newly diagnosed T2DM to distinguish early versus established alterations in myokine–metabolic interactions.

Overall, the color scale ranges from light green (weak negative correlation) to orange/yellow (weak-to-moderate positive correlation), with white tones representing negligible or non-significant relationships.

**Figure 5:**
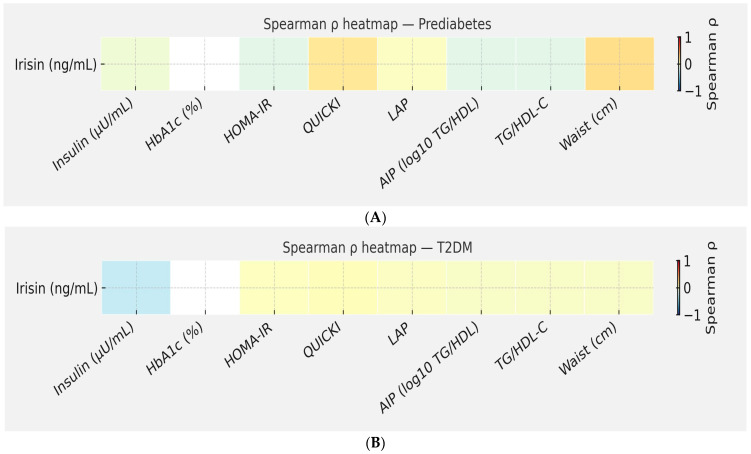
Spearman correlation heatmaps showing associations between serum irisin and metabolic parameters in prediabetic (PreDM) (**A**) and type 2 diabetes (T2DM) (**B**) participants. (**A**) The color scale represents the strength and direction of Spearman correlation coefficients (*ρ*): green tones: negative correlations (*ρ* < 0); white tones: near-zero or non-significant correlations (*ρ* ≈ 0); orange to yellow tones: positive correlations (*ρ* > 0). In the PreDM group, irisin displayed moderate inverse correlations with lipid-related indices—LAP, AIP, and TG/HDL-C—indicating that higher lipid accumulation and atherogenicity are associated with lower serum irisin levels even before diabetes onset. The relationships with HOMA-IR and QUICKI were weaker and did not reach significance, suggesting that in the PreDM state, irisin variability is more strongly driven by dyslipidemia than by insulin sensitivity. No relevant associations were observed between irisin and HbA1c, insulin, or waist circumference. (**B**) The heatmap shows Spearman correlation coefficients (*ρ*) between serum irisin and metabolic parameters in participants with newly diagnosed T2DM. The color scale represents the direction and magnitude of Spearman correlation coefficients (*ρ*): blue tones: weak negative correlations (*ρ* < 0); white tones: near-zero correlations (*ρ* ≈ 0); yellow tones: weak-to-moderate positive correlations (*ρ* > 0). In the T2DM group, irisin correlated negatively with insulin (*ρ* = −0.316), HOMA-IR (*ρ* = −0.384), LAP, and atherogenic indices (AIP and TG/HDL-C), while positively correlating with QUICKI, consistent with its proposed role in enhancing insulin sensitivity and mitigating lipid-related risk. These findings highlight that, in overt T2DM, lower irisin levels are associated with higher insulin resistance and an adverse lipid phenotype. In contrast, correlations with HbA1c and waist circumference remain weak, suggesting a secondary role of chronic glycemia and anthropometry in determining circulating irisin concentrations. Abbreviations: HbA1c, glycated hemoglobin; HOMA-IR, homeostatic model assessment of insulin resistance; QUICKI, quantitative insulin sensitivity check index; LAP, lipid accumulation product; AIP, atherogenic index of plasma; TG/HDL-C, triglyceride-to-HDL cholesterol ratio.

In PreDM subjects (Table 12, Figure 5A), irisin exhibits mild positive correlations with QUICKI, LAP, and waist circumference, as shown by light orange–yellow shading. These associations suggest that higher irisin levels may correspond to slightly improved insulin sensitivity (as measured by QUICKI) and modestly increased adiposity indices (LAP, waist), reflecting compensatory secretion from muscle tissue in early dysmetabolic states. Conversely, insulin, HbA1c, HOMA-IR, and AIP show very weak or absent correlations (light green to near-white cells), indicating that in prediabetes, irisin’s variation is not tightly linked to hyperinsulinemia, glycemic control, or plasma lipid atherogenicity.

Thus, in PreDM subjects, irisin correlates weakly and inconsistently with metabolic parameters, implying a transitional stage in which muscle–adipose signaling remains partially preserved but not yet disrupted as in overt diabetes.

In T2DM patients (Table 13, Figure 5B), most relationships appear in light yellow tones, indicating weak positive correlations (*ρ* between +0.100 and +0.300). These patterns suggest that as T2DM progresses, irisin levels are modestly correlated with indices of lipid accumulation (LAP, TG/HDL-C, AIP) and anthropometric adiposity (waist circumference), consistent with irisin’s role as a myokine linked to energy expenditure and fat metabolism.

Notably, the light blue shade observed for insulin and, to a lesser extent, HbA1c reflects a weak inverse correlation (*ρ* ≈ −0.100 to −0.200), implying that higher irisin concentrations may be slightly associated with lower fasting insulin levels and better glycemic control. These inverse trends, though small, could represent a residual protective or compensatory effect of irisin under chronic metabolic stress.

Overall, the correlation pattern in T2DM differs from that in PreDM, showing greater coupling of irisin with lipid and adiposity measures and a weaker link with insulin resistance. This suggests that in overt diabetes, irisin’s regulatory role is increasingly driven by lipid metabolism rather than glucose regulation.

Taken together, these findings suggest that circulating irisin is closely linked to lipid metabolism and insulin sensitivity, with its associations shifting from predominantly lipid-related in prediabetes to both lipidic and insulin-resistance–related in overt T2DM. This pattern supports the hypothesis that irisin may play a compensatory role in early dysmetabolic stages, which diminishes as insulin resistance and adipose dysfunction progress.

#### Interpretation of Irisin–Metabolic Correlations After Multiple Comparison Adjustment

To account for potential inflation of error due to multiple comparisons, correlation analyses between serum irisin and metabolic parameters were further adjusted using the Benjamini–Hochberg false discovery rate (FDR) correction. Separate analyses were performed for prediabetic and T2DM participants to assess whether group-specific association patterns persisted after statistical adjustment. After FDR correction, no correlations between circulating irisin and the evaluated metabolic parameters remained statistically significant in either the PreDM or T2DM groups (Table 14).

In PreDM subjects, the strongest unadjusted association was an inverse trend between irisin and HbA1c (*ρ* = −0.319, *p* = 0.027; *p* (FDR) = 0.217), suggesting that lower irisin levels may be weakly associated with early glycemic deterioration. However, this association did not withstand FDR correction, and other relationships with insulin resistance indices (HOMA-IR, QUICKI) or lipid-derived markers (LAP, AIP, TG/HDL-C) were not significant.

In T2DM patients, the correlation coefficients were uniformly weak and non-significant, indicating that once diabetes is established, serum irisin levels show no consistent association with insulin resistance, glycemic control, or lipid indices. These findings suggest that the apparent associations observed in unadjusted analyses are likely due to random variation and that irisin is not independently correlated with individual metabolic indices after correction for multiple testing.

## 3. Discussion

This study investigated the associations among circulating irisin, insulin resistance indices, lipid-derived composite markers, and the HTGW phenotype in individuals with PreDM and newly diagnosed T2DM. Our results demonstrated that serum irisin levels were significantly lower in T2DM than in PreDM, consistent with the hypothesis that irisin secretion declines with advancing metabolic dysfunction [16,17,18,44]. Although the differences in lipid parameters (LDL-C, HDL-C, and derived indices such as AIP and LAP) were pronounced between groups, multiple regression and logistic analyses revealed that these associations are not independent but rather reflect overlapping metabolic pathways linking insulin resistance, adiposity, and lipid accumulation [45,46].

The observed reduction in circulating irisin levels in T2DM agrees with most published studies reporting decreased irisin concentrations in diabetes and metabolic syndrome [47,48,49,50,51]. Studies reported significantly lower irisin levels in newly diagnosed T2DM patients vs. controls [17,18,25]. Other population studies have confirmed this trend, with lower irisin levels independently predicting poor insulin sensitivity [20,21].

However, results in prediabetes remain heterogeneous. Some studies reported higher irisin concentrations at early dysglycemic stages, possibly reflecting a compensatory mechanism [45,52], whereas others observed a steady decline preceding overt diabetes [47,53]. Our finding of higher irisin in PreDM than T2DM, but without independent predictive power after adjustment, supports the compensatory hypothesis: skeletal muscle may transiently increase irisin secretion in early metabolic stress, but this response diminishes with progressive insulin resistance and adipocyte dysfunction [54].

Regarding lipid metabolism, we found negative correlations between irisin and TG/HDL-C, AIP, and LAP in both groups, consistent with prior studies linking lower irisin to a more atherogenic lipid profile [50,55,56,57,58,59]. For instance, Rana et al. [52] observed that elevated plasma irisin in T2DM is associated with indices of adiposity, and Mathia et al. (2023) [55] suggested that body mass index directly influences plasma irisin levels [50]. These consistent inverse patterns suggest that irisin is more closely linked to lipid metabolism and visceral adiposity than to glycemia per se.

The multiple linear regression models clarified that irisin variation in PreDM is weakly explained by glycemic or lipid markers. Both AIP- and LAP-based models were nonsignificant, indicating high interdependence among predictors. This reflects the multifactorial regulation of irisin secretion and the presence of metabolic compensations in early dysglycemia [48,51,59]. Multicollinearity among insulin resistance indices (VIF > 20) indicates overlapping variance among insulin, HOMA-IR, and QUICKI, as reported in other metabolic modeling studies [49,50]. In contrast, T2DM models yielded modest but interpretable associations. In the AIP and LAP models, WC was a negative independent predictor of irisin (β = –1.71, *p* = 0.018; β = –3.01, *p* = 0.004), and LAP showed a borderline positive association (β = 0.58, *p* = 0.092). This suggests that visceral adiposity plays a dominant role in lowering irisin, consistent with findings from Crujeiras et al. [60] and Oelmann et al. [50], who both linked irisin decline to increased central adiposity [21].

Taken together, these findings indicate that irisin variability is best explained by integrated metabolic stress rather than isolated insulin or lipid parameters. This pattern supports the hypothesis that, in T2DM, reduced irisin levels reflect impaired muscle–adipose cross-talk and the loss of its compensatory endocrine function [20,45]. The logistic regression analysis further supported these observations. In the combined model (χ^2^(7) = 33.42, *p* < 0.001; Nagelkerke R^2^ = 0.317), irisin was not an independent predictor of HTGW (OR = 0.94, *p* = 0.814), whereas HbA1c was (OR = 1.42, *p* = 0.048). Age (OR = 0.94, *p* = 0.015) and sex (OR = 0.12, *p* < 0.001) were also significant factors, reflecting hormonal and adipose-distribution influences consistent with previous literature [47,56].

When stratified, the PreDM model (χ^2^(6) = 15.87, *p* = 0.014; AUC = 0.794, 95% CI 0.674–0.883) showed a nonsignificant protective trend for irisin (OR = 0.72, *p* = 0.362), whereas in T2DM (χ^2^(6) = 19.56, *p* = 0.003; AUC = 0.813, 95% CI 0.725–0.893) the same trend disappeared (OR = 1.08, *p* = 0.810). The repeated emergence of HbA1c as an independent predictor in both strata underscores the central role of glycemic burden in HTGW development [54,58]. This is in line with Kahla et al. and Akyol Tulgar et al., who both emphasized the contribution of chronic dysglycemia and lipid accumulation to the HTGW phenotype [47,53].

Collectively, these data suggest that, while irisin interacts with lipid and adiposity markers, it does not independently predict the HTGW phenotype after accounting for major glycemic determinants. This pattern reinforces the concept of “irisin resistance” in advanced metabolic states [21,50,61].

Building upon the regression findings, the comparative analysis of ROC models further elucidated irisin’s contribution to metabolic risk discrimination. The global logistic regression model, integrating standardized irisin, HbA1c, insulin, BMI, age, sex, and glycemic group, demonstrated strong discriminative capacity for identifying individuals with the HTGW phenotype (AUC = 0.806). This AUC value indicates excellent classification performance, even after excluding waist circumference to avoid overadjustment. When the models were stratified by glycemic status, both retained high discriminative power (AUC = 0.794) in PreDM and in T2DM (AUC = 0.813), with overlapping confidence intervals, suggesting comparable model accuracy across disease stages. The slightly higher AUC in T2DM may reflect the additive contribution of chronic hyperglycemia and insulin resistance to the combined dyslipidemic–adiposity phenotype.

Notably, despite irisin’s lack of statistical independence in logistic regression, its inclusion contributed to stable AUC values, suggesting a modulatory rather than primary predictive role. These findings align with the growing body of literature suggesting that irisin is a secondary integrative marker that reflects composite metabolic stress rather than a direct determinant of single metabolic outcomes [17,50,51,52]. The consistent discriminative accuracy across glycemic stages (AUC ≈ 0.8) underscores the model’s robustness. At the same time, the similar performance between PreDM and T2DM implies that the HTGW phenotype is established early in dysglycemia, before overt diabetes develops [53,54].

The biological mechanisms underpinning these associations highlight irisin’s complex endocrine role in muscle–adipose–liver communication. Irisin is produced by proteolytic cleavage of the transmembrane protein FNDC5, under transcriptional control of peroxisome proliferator-activated receptor gamma coactivator 1-alpha (PGC-1α), a central regulator of mitochondrial biogenesis and oxidative metabolism [62,63].

Once secreted, irisin stimulates the “browning” of white adipose tissue, enhancing thermogenesis by upregulating uncoupling protein 1 (UCP1) and promoting increased energy expenditure [11,21,60]. Through activation of the AMP-activated protein kinase (AMPK) pathway, irisin improves glucose uptake in skeletal muscle, augments insulin sensitivity, and reduces hepatic gluconeogenesis [46,58]. In vitro studies demonstrate that irisin upregulates glucose transporter type 4 (GLUT4) and activates PI3K/AKT signaling, directly improving insulin signaling cascades [21,64].

In the context of lipid metabolism, irisin reduces triglyceride accumulation in hepatocytes, inhibits lipogenesis, and enhances β-oxidation, thereby improving plasma lipid profiles [48,50]. The observed inverse correlations with TG/HDL-C, AIP, and LAP in our study support these mechanisms.

The concept of “irisin resistance”, proposed in obesity and advanced diabetes, suggests that chronic metabolic stress may impair irisin signaling, leading to elevated secretion but diminished activity [20,51]. In our cohort, however, the decline of circulating irisin from PreDM to T2DM, together with its weak association with insulin resistance indices, points toward reduced myokine production rather than compensatory hypersecretion—potentially reflecting impaired PGC-1α activation and skeletal muscle mitochondrial dysfunction.

The consistent association between lower irisin and adverse metabolic indices suggests potential clinical applications for risk stratification and early detection. In particular, the coexistence of low irisin levels and elevated LAP or AIP could help identify individuals at high cardiometabolic risk even before overt hyperglycemia develops [21,52].

From a translational standpoint, interventions that increase irisin expression—such as structured physical activity, resistance training, and caloric restriction—could be valuable adjuncts in metabolic disease prevention [11,60,63]. Several studies have demonstrated that exercise-induced increases in irisin improve insulin sensitivity and endothelial function in both animal and human models [46,48]. Furthermore, pharmacological activation of PGC-1α/FNDC5 signaling has been proposed as a novel therapeutic approach to enhance energy expenditure and mitigate metabolic dysfunction [11,58].

However, our regression models indicate that irisin alone is unlikely to serve as a stand-alone biomarker. Instead, it may be most effective when integrated into composite panels combining glycemic, lipidic, and anthropometric markers, thereby enhancing predictive accuracy for phenotypes such as HTGW and metabolic syndrome.

### Limitations

Several limitations must be acknowledged. First, the cross-sectional design precludes causal inference. Longitudinal studies are necessary to confirm whether declining irisin precedes or merely reflects metabolic deterioration. Second, the sample size, though adequate for statistical modeling, may limit generalizability across populations with diverse ethnic and lifestyle backgrounds. Third, despite rigorous analytical procedures, the absence of standardized ELISA kits for irisin measurement remains a significant limitation, as previously highlighted by several systematic reviews [8,51]. Variability in antibody specificity and assay sensitivity can introduce bias across studies. Unmeasured confounders—such as physical activity level, muscle mass, or dietary intake—may influence circulating irisin concentrations and were not assessed here. Future research should incorporate body composition analysis and cardiorespiratory fitness metrics to clarify the muscle-derived contribution to circulating irisin.

Although our design did not include a healthy normoglycemic control group, several studies spanning the glucose tolerance continuum suggest that circulating irisin is highest in NGT and declines as dysglycemia progresses. In a cohort explicitly comparing NGT, prediabetes, and type 2 diabetes with matching for age, sex, and BMI, Assyov et al. reported a stepwise reduction in median irisin from NGT to prediabetes and then to T2D [40,65].

A similar gradient was reported by Duran et al. in sedentary women, with lower irisin in impaired fasting glucose plus impaired glucose tolerance and in T2DM than in NGT [66]. This pattern is directionally consistent with our finding of lower irisin in newly diagnosed T2DM than in prediabetes (median 140.4 vs. 230.7 ng/mL). However, absolute irisin concentrations vary substantially across studies because of preanalytical factors and between kit and assay differences, so comparisons across cohorts are best interpreted qualitatively (higher vs. lower), not by raw values.

Beyond individual cohorts, pooled evidence generally supports lower irisin in T2DM versus healthy controls. For example, Zhang et al. combined a case–control study with a meta-analysis and found circulating irisin to be lower in T2DM than in controls in the pooled estimate [16]. Earlier clinical studies in new onset T2DM likewise reported reduced irisin relative to normal glucose tolerant controls [16]. At the same time, the literature remains heterogeneous, with some studies reporting unchanged or even higher irisin in early dysglycemia, which has been interpreted as a possible compensatory response and also reflects methodological variability [67]. In one of our recent studies, we observed that PreDM and T2DM can have similar BMI and triglycerides yet differ in glycemic burden and in non-insulin-based insulin resistance indices, such as METS-IR and TyG, which track closely with central adiposity measures (BRI, AVI, WWI) [68]. Such phenotyping may help contextualize between study differences in observed irisin patterns across the dysglycemia spectrum.

A potential source of selection bias is the large exclusion in the T2DM arm. Of 180 consecutively screened patients with newly diagnosed T2DM, 90 were excluded because chronic microvascular complications were already present (peripheral polyneuropathy n = 45, diabetic kidney disease n = 25, retinopathy n = 20). This restriction was deliberate, we aimed to characterize early, uncomplicated T2DM and to avoid confounding from complication related inflammation, reduced mobility, and impaired renal function, all of which could plausibly affect circulating irisin. However, it also narrows the clinical spectrum toward “milder” or earlier diagnosed cases, so our estimates may not generalize to the broader T2DM population, particularly patients with longer undetected disease and established complications. If irisin differs systematically in patients with microvascular disease, excluding them could shift the group distribution and change the apparent PreDM vs. T2DM contrast. Residence is another possible confounder. The T2DM group included more urban participants than the PreDM group (urban 57 of 90 vs. 26 of 48, *p* = 0.294). Even when the difference is not statistically significant, urban or rural residence can act as a proxy for lifestyle and access factors relevant to irisin, such as habitual physical activity, occupational exertion, diet, socioeconomic status, and healthcare utilization. Because these were not incorporated as covariates in the main models, residual confounding remains possible, and it may contribute to between-group differences attributed to glycemic status. Future work would benefit from tighter matching on residence, plus adjustment for quantified physical activity, body composition, and dietary patterns, and from stratified analyses by urban or rural status.

A limitation of this study is that circulating irisin was measured using a commercial antibody-based ELISA. Although the assay showed acceptable analytical precision in our hands (intra-assay and inter-assay CVs < 10%) and the manufacturer reports good recovery and dilutional linearity, irisin measurement remains methodologically challenging. Concerns have been raised about antibody specificity and between-kit variability and reported absolute concentrations can differ substantially across studies depending on the assay used. Therefore, our findings are best interpreted as relative differences within this cohort rather than definitive absolute irisin concentrations. Future work could confirm concentrations using targeted mass spectrometry approaches with stable isotope-labeled standards, which have been used as an antibody-independent quantification strategy [69,70,71].

Prospective and interventional studies evaluating exercise- or diet-induced changes in irisin and their effects on lipid indices (AIP, LAP) and HTGW phenotypes would further elucidate causality. Molecular studies examining irisin receptor signaling and downstream metabolic pathways may also help clarify whether irisin primarily acts as a biomarker or as a metabolic effector.

## 4. Materials and Methods

### 4.1. Patient Selection

Over 6 months, we conducted a retrospective cohort study at a single university hospital in Craiova, Dolj, Romania. This study enrolled 180 consecutive patients with newly diagnosed T2DM. Additionally, 100 PreDM patients who met the inclusion criteria—based on age, gender ratio, and urban/rural location—served as the control group. The study complied with the Declaration of Helsinki and was approved by the Ethics Committee of the Clinical Municipal Hospital Filantropia Craiova (no. 887/15 January 2024).

We selected individuals aged 18 or older with newly diagnosed T2DM from the Diabetes, Nutrition, and Metabolic Diseases Departments of the Clinical Municipal Hospital Filantropia Craiova. All participants voluntarily participated in the study after providing informed consent.

The study excluded patients with chronic microvascular complications of T2DM, including diabetic peripheral polyneuropathy, diabetic kidney disease, and diabetic retinopathy. Additionally, exclusion criteria involved patients under 18, pregnant women, individuals with type 1 diabetes, and those who had an acute infection or inflammatory disease within the past month. Patients with ongoing infections, inflammatory conditions, or cancer were also excluded from the study.

We examined 90 patients newly diagnosed with T2DM and 48 PreDM patients (See Section 4.2). After applying several exclusion criteria, 90 patients with T2DM were lost to follow-up for various reasons: diabetic peripheral polyneuropathy (n = 45), diabetic kidney disease (n = 25), and diabetic retinopathy (n = 20).

PreDM was characterized in multiple ways, including: (1) a diagnosis by a healthcare professional; (2) an HbA1c level from 5.7% to just below 6.5%; (3) an FPG level between 5.6 mmol/L and 7.0 mmol/L; or (4) an OGTT result from 7.8 mmol/L to 11.0 mmol/L [3]. T2DM patients were diagnosed based on at least one of the following criteria: a medical diagnosis confirmed by the patient’s healthcare providers, an HbA1c level above 6.5%, an FPG level of 7.0 mmol/L or higher, a random blood glucose level of 11.1 mmol/L or higher, or a two-hour blood glucose level exceeding 11.1 mmol/L after an OGTT. Additionally, a random glucose measurement combined with classic hyperglycemic symptoms, such as polyuria, polydipsia, and unexplained weight loss, or hyperglycemic crises [3], may also indicate diabetes.

Data on physical measurements, medical conditions, lab test results, personal and lifestyle factors were gathered via an interview questionnaire. Demographic variables encompassed age, gender, monthly household income, and educational attainment. Factors related to lifestyle and health, including history of smoking and alcohol use, family history of diseases such as high blood pressure, diabetes, and heart disease, and the amount of time spent engaging in moderate physical activity each week, were recorded for analysis.

### 4.2. Definitions of Hypertriglyceridemic-Waist (HTGW) Phenotypes

Based on the Standards of Care in Diabetes—2025 of the American Diabetes Association Professional Practice Committee [3], using WC (<90 cm in males or <80 cm in females) and TG level (≤150 mg/dL (1.7 mmol/L)) as thresholds, the subjects were divided into four HTGW phenotypes [72]:

- NTNW: normal TG level (≤150 mg/dL (1.7 mmol/L)) and normal WC (<90 cm in males or <80 cm in females);
- NTEW: normal TG level (≤150 mg/dL (1.7 mmol/L)) and enlarged WC (≥90 cm in males or ≥80 cm in females);
- ETNW: elevated TG level (>150 mg/dL (1.7 mmol/L)) and normal WC (<90 cm in males or <80 cm in females);
- HTGW: elevated TG level (>150 mg/dL (1.7 mmol/L)) and enlarged WC (≥90 cm in males or ≥80 cm in females).

After applying these criteria, we identified the following cohorts based on metabolic phenotypes:

- PreDM cohort: the NTNW (n = 50), NTEW (n = 20), ETNW (n = 13), and HTGW (n = 15) phenotypes;
- T2DM cohort: the NTEW (n = 40), ETNW (n = 21), and HTGW (n = 29) phenotypes.

Given the absence of patients with NTNW in the T2DM cohort, our comparative analysis was limited to the NTEW, ETNW, and HTGW phenotypes. Therefore, we excluded 50 patients with the NTNW from the PreDM. Consequently, 90 patients with T2DM and 48 with PreDM completed the study and were included in the final analysis.

### 4.3. Evaluation of Various Indices of Insulin Resistance and Lipid-Related Risk Factors (HOMA-IR, QUICKI, TG/HDL-C, AIP, and LAP)

To evaluate insulin resistance and the metabolic profile, we used the formulas in Table 15.

### 4.4. Laboratory Investigations

Following the acquisition of the anthropometric data, we performed more thorough evaluations of the subjects in the lab.

#### 4.4.1. Sample Collection

Each patient provided two blood samples, collected in separate tubes, as part of the biological sampling process.

- Two additive-free tubes containing roughly 5 mL of each patient’s venous blood (Becton Dickinson Vacutainer, Franklin Lakes, NJ, USA). Following standard processing procedures, blood samples were centrifuged in a Hermle centrifuge (Hermle AG, Gosheim, Baden-Württemberg, Germany) at 3000× *g* for 10 min, within 4 h of collection, after clotting. To guarantee sample preservation, the resultant serum from a single tube was aliquoted into pre-labeled vials, carefully sealed to prevent contamination, and stored at regulated temperatures between −20 °C and −80 °C. Freeze-thaw cycles were strictly avoided to preserve specimen integrity. Frozen serum samples were passively thawed to room temperature before analysis. Immunological studies were conducted using these aliquots. Biochemical analyses were conducted using the serum from the second tube.
- Additionally, a complete blood count (CBC) was performed using peripheral venous blood obtained in ethylene-diamine-tetra-acetic acid (EDTA) vacutainer tubes (Becton Dickinson Vacutainer, Franklin Lakes, NJ, USA). The MINDRAY BC-6800 (Mindray, Shenzhen, China) was used to successfully analyze five parameters to develop an extended leukocyte differential using flow cytometry. With this method, we successfully discovered and described a variety of hemoleucogram markers.

#### 4.4.2. Biochemical Investigations

Using the ARCHITECT c4000 clinical chemistry analyzer (Abbott Laboratories, Abbott Park, IL, USA), which uses photo-metric, enzymatic, and potentiometric techniques depending on the analyte, biochemical parameters were evaluated. The manufacturer’s normal operating procedures were followed for the analysis of serum or plasma samples. Every measurement was carried out in accordance with the equipment’s calibration and quality control protocols.

### 4.5. Immunological Assessment

Serum concentrations of Insulin and Irisin were measured using the Enzyme-Linked Immunosorbent Assay (ELISA) kits from Elabscience (Houston, TX, USA) in the Immunology Laboratory of the University of Medicine and Pharmacy of Craiova: Insulin (Cat. No.: E-EL-H2665; Sensitivity: 0.47 μIU/mL; Detection Range: 0.78–50 μIU/mL; Specificity: No significant cross-reactivity or interference between Human INS and analogs was observed—https://789.bio/ea/mPSqr9 (accessed on 10 November 2025); Intra-/Inter-Assay CV (%): Coefficient of variation is <10%; https://www.elabscience.com/p/human-ins-insulin-elisa-kit--e-el-h2665, (accessed on 10 November 2025), and Irisin (Cat. No.: E-EL-H5735; Sensitivity: 46.88 pg/mL; Detection Range: 78.13–5000 pg/mL; Specificity: No significant cross-reactivity or interference between Human Irisin and analogs was observed—https://789.bio/ea/v5ejH8 (accessed on 10 November 2025); Intra-/Inter-Assay CV (%): Coefficient of variation is <10%; https://www.elabscience.com/p/human-irisin-elisa-kit--e-el-h5735 (accessed on 10 November 2025); recovery 80–120%). We applied the competitive ELISA principle using a standard optical analyzer, the Asys Expert Plus UV G020 150 Microplate Reader (ASYS Hitech GmbH, Eugendorf, Austria), operating at 450 nm, in accordance with the manufacturer’s instructions and recommended methods.

### 4.6. Statistical Analysis

Data were analyzed using GraphPad Prism 10.6.1 (892) (GraphPad Software, San Diego, CA, USA). Continuous variables were tested for normality using the Shapiro–Wilk test and presented as mean ± SD or median [IQR], as appropriate.

Following a two-way ANOVA assessing the effects of glycemic status and HTGW phenotype, pairwise post hoc comparisons were conducted using the Tukey test with Holm–Bonferroni correction when overall effects were significant. Adjusted *p*-values < 0.05 were considered statistically significant. The analysis identified group-specific differences in metabolic markers, depending on the interaction between phenotype and diabetes status.

Bivariate associations between circulating irisin and metabolic variables were evaluated using Spearman’s rank correlation coefficients (ρ) due to the non-normal distribution of several parameters. Correlations were calculated separately for PreDM and T2DM participants. To account for multiple testing, *p*-values were adjusted using the Benjamini–Hochberg false discovery rate (FDR) correction (q = 0.05). Both raw and FDR-adjusted *p*-values were reported.

To explore the independent relationships between irisin and metabolic indices, multiple linear regression (MLR) analyses were performed separately for PreDM and T2DM groups. The dependent variable was serum irisin concentration (ng/mL). Predictors initially included lipid ratios (AIP, TG/HDL-C), adiposity markers (LAP, waist circumference), glycemic indices (HbA1c, HOMA-IR, QUICKI), and demographic covariates (age, sex): *Model* 1 *(AIP)*: AIP (log_10_ TG/HDL), Insulin, HbA1c, HOMA-IR, QUICKI, TG/HDL-C, Waist, Age, Sex; *Model* 2 *(LAP)*: LAP, Insulin, HbA1c, HOMA-IR, QUICKI, TG/HDL-C, Waist, Age, Sex. The linearity assumption and absence of multicollinearity were verified using residual plots and the variance inflation factor (VIF) (threshold < 5). Both raw and standardized β coefficients were computed to enable comparison of effect sizes across predictors. Because several predictors exhibited conceptual overlap or high collinearity (e.g., HOMA-IR vs. insulin, or AIP vs. TG/HDL-C and LAP), final regression analyses used parsimonious model specifications to improve interpretability and model stability. AIP and LAP were tested in separate models as representative composite indices of atherogenic dyslipidemia and visceral adiposity, respectively. HbA1c, age, and sex were retained as mandatory covariates, whereas redundant lipid or insulin resistance variables were excluded. This approach minimized overfitting and provided more reliable estimates of the independent contributions of each metabolic domain to circulating irisin variability.

Logistic regression analysis was performed to assess the independent predictors of the HTGW phenotype. The dependent variable was HTGW status (0 = non-HTGW, 1 = HTGW): global model—*HTGW (yes/no)* = *Irisin (standardized)* + *Age (y)* + *Sex (Male* = 1*)* + *BMI* (kg/m^2^) + *group (T2DM* = 1*)* + *HbA1c (*%*)* + *insulin* (µU/mL). Separate multivariate models were fitted for the entire cohort and stratified by glycemic category (PreDM and T2DM). Independent variables included standardized irisin (per 1 SD), age, sex, BMI, HbA1c, and insulin concentrations. The logistic models were estimated using the maximum likelihood method. The likelihood-ratio Chi-square (χ^2^) statistic tests the overall significance of each model by comparing it with a null (intercept-only) model. A significant χ^2^ (*p* < 0.05) indicated that the predictors jointly improved model fit. The Nagelkerke R^2^ coefficient was used as a pseudo-R-squared index to approximate the proportion of variance in HTGW status explained by the model, providing an indicator of its explanatory power (values closer to 1 indicate a stronger fit). Model calibration was evaluated using the Hosmer–Lemeshow goodness-of-fit test, which compares observed and predicted frequencies of HTGW across deciles of risk. A non-significant Hosmer–Lemeshow *p*-value (*p* > 0.05) denotes adequate model calibration, whereas a significant value indicates potential misfit between predicted and observed outcomes. Model discrimination was assessed through receiver operating characteristic (ROC) curve analysis, with the area under the curve (AUC) and 95% confidence intervals (CI) calculated via bootstrap resampling (2.000 iterations). ROC curves were compared between PreDM and T2DM groups to evaluate the consistency of the models’ predictive performance.

## 5. Conclusions

In summary, this study demonstrates that circulating irisin levels progressively decline from PreDM to newly diagnosed T2DM and are inversely related to insulin resistance and lipid atherogenicity. While bivariate analyses revealed modest correlations between irisin, HOMA-IR, AIP, and LAP, multiple regression models indicated that these associations are largely dependent on visceral adiposity and glycemic status. In T2DM, waist circumference was the only independent negative predictor of irisin, suggesting that chronic central obesity suppresses myokine secretion. Logistic regression further showed that HbA1c, age, and sex, rather than irisin, are the main predictors of the HTGW phenotype, although the inclusion of irisin slightly improved model discrimination. Collectively, these findings support the concept that irisin acts as an adaptive biomarker in early dysmetabolism but becomes downregulated under sustained metabolic stress, reflecting the shift from compensatory to maladaptive energy regulation. Integrating irisin with composite lipid and adiposity indices, such as AIP and LAP, may enhance cardiometabolic risk stratification, particularly in prediabetic individuals, and highlight its potential as an early marker of the HTGW phenotype. In conclusion, our findings emphasize the importance of a neuro-metabolic approach in assessing PreDM and newly diagnosed T2DM, moving beyond glucose-focused models. Larger prospective studies are needed to validate neurotransmitters as biomarkers and to examine their potential in precision prevention and treatment strategies. 

## Figures and Tables

**Table 1:** Baseline characteristics of study participants (mean ± SD or median [IQR]).

Variable	PreDM (n = 48)	T2DM (n = 90)	*p* Value
Age (years) (mean ± SD)	54.25 ± 11.81	57.18 ± 10.61	0.155
Sex (Male/Female)	24/24	51/39	0.454
Areas (Rural/Urban)	22/26	33/57	0.294
Weight (kg) (median [IQR])	73.75 [66.25–92.00]	87.00 [75.75–102.00]	0.003
Height (cm) (mean ± SD)	162.00 ± 8.75	168.20 ± 10.25	0.001
Waist (cm) median [IQR])	97.00 [88.00–102.00]	109.00 [105.78–117.50]	<0.0001
Hip (cm) (mean ± SD)	105.90 ± 11.98	111.43 ± 11.49	0.010
MAP (mmHg) (mean ± SD)	88.81 ± 8.54	91.52 ± 8.32	0.076
BMI (kg/m^2^) (median [IQR])	29.22 [24.91–36.48]	31.08 [26.87–35.51]	0.3548
HbA1c (%) (mean ± SD)	5.98 ± 0.22	9.03 ± 1.98	<0.0001
FPG (mg/dL) (median [IQR])	104.00 [97.75–110.20]	156.00 [138.00–185.30]	<0.0001
Insulin (µU/mL) (median [IQR])	3.28 [2.47–4.66]	49.82 [43.70–51.61]	<0.0001
TC (mg/dL) (median [IQR])	200.00 [154.80–230.50]	189.50 [157.50–219.30]	0.439
TG (mg/dL) (median [IQR])	154.50 [101.30–188.10]	156.00 [137.00–222.50]	0.026
HDL-C (mg/dL) (median [IQR])	49.61 [41.20–62.00]	45.00 [37.00–51.25]	0.006
LDL-C (mg/dL) (mean ± SD)	112.60 ± 38.66	147.60 ± 31.90	<0.0001
HOMA-IR (median [IQR])	0.85 [0.57–1.19]	18.33 [15.77–21.96]	<0.0001
QUICKI (median [IQR])	0.17 [0.16–0.18]	0.11 [0.11–0.12]	<0.0001
hs-CRP (mg/L) (median [IQR])	19.25 [15.08–36.30]	18.70 [10.18–49.78]	0.787
Simple Atherogenic Ratios			
TG/HDL-C (median [IQR])	2.82 [2.33–3.56]	3.94 [2.98–5.33]	<0.0001
AIP (log10 TG/HDL) (median [IQR])	0.45 [0.37–0.55]	0.60 [0.47–0.73]	<0.0001
Adiposity Accumulation/Myokine			
LAP (median [IQR])	50.17 [39.33–70.07]	86.94 [64.96–110.30]	<0.0001
Irisin (ng/mL) (median [IQR])	230.70 [210.40–249.90]	140.40 [118.20–178.60]	<0.0001
Hypertriglyceridemic-waist (HTGW) phenotype, *n* (%)			
Normal TG and enlarged WC (NTEW)	20 (41.67%)	40 (44.44%)	0.752
Elevated TG and normal WC (ETNW)	13 (27.08%)	21 (23.33%)	0.624
Increased TG and enlarged WC (HTGW)	15 (31.25%)	29 (32.23%)	0.920

MAP: mean arterial pressure; BMI: body mass index; HbA1c: glycosylated hemoglobin A1c; FPG: fasting plasma glucose; TC: total cholesterol; TG: total triglycerides; HDL-C: high-density lipoprotein cholesterol; LDL-C: low-density lipoprotein cholesterol; HOMA-IR: Homeostatic Model Assessment of Insulin Resistance; QUICKI: Quantitative Insulin Sensitivity Check Index; AIP: atherogenic index of plasma; LAP: lipid accumulation product; hs-CRP: high-sensitivity C-reactive protein; WC: waist circumference.

**Table 2:** Comparation of Irisin and Metabolic Parameters Levels According to Gender in the PreDM and T2DM groups.

Parameters (Median [IQR])		PreDM		T2DM		
	Male (*n* = 24)	Female (*n* = 24)	*p* Value	Male (*n* = 51)	Female (*n* = 39)	*p* Value
Irisin (ng/mL)	230.00 218.00–413.75	238.50 207.50–429.00	0.915	149.00 128.00–236.00	167.00 135.00–231.00	0.867
Insulin (µU/mL)	3.05 2.48–4.77	3.69 2.25–4.30	0.805	49.84 45.12–51.31	49.80 42.07–52.28	0.995
HOMA-IR	0.76 0.58–1.28	0.93 0.56–1.18	0.724	18.48 15.69–22.95	17.93 15.93–20.96	0.554
QUICKI	0.18 0.16–0.19	0.17 0.16–0.19	0.735	0.11 0.10–0.12	0.12 0.10–0.14	0.626
TG/HDL-C	2.69 2.11–3.29	3.17 2.55–3.66	0.077	4.04 2.98–6.04	3.80 3.02–4.72	0.337
AIP (log10 TG/HDL)	0.43 0.33–0.52	0.51 0.40–0.56	0.057	0.61 0.47–0.78	0.58 0.48–0.67	0.307
LAP	40.73 35.45–49.83	67.05 51.29–88.82	<0.0001	76.55 53.11–96.73	96.94 79.66–117.40	0.004

HOMA-IR: Homeostatic Model Assessment of Insulin Resistance; QUICKI: Quantitative Insulin Sensitivity Check Index; AIP: atherogenic index of plasma; LAP: lipid accumulation product.

**Table 3:** Comparing the Irisin and Metabolic Parameters Levels According to Hypertriglyceridemic-waist (HTGW) phenotype in the PreDM and T2DM Groups.

**Parameter** **Median [IQR]**	***PreDM***					
	**NTEW** **(** ***n*** **= 20)**	**ETNW** **(** ***n*** **=13)**	**HTGW** **(** ***n*** **= 15)**	**Factor**	**F** **(DFn, DFd)**	***p*** **Value**
Insulin (µU/mL)	3.58 1.87–5.31	3.27 2.40–4.41	3.19 2.86–4.01	Row factor	F (19,26) = 0.531	0.921
				Column factor	F (2,26) = 0.182	0.835
HOMA-IR	0.91 0.47–1.38	0.80 0.48–1.07	0.89 0.66–1.19	Row factor	F (19,26) = 0.543	0.913
				Column factor	F (2,26) = 1.001	0.381
QUICKI	0.17 0.16–0.19	0.17 0.16–0.19	0.17 0.16–0.18	Row factor	F (19,26) = 0.474	0.951
				Column factor	F (2,26) = 0.516	0.603
TG/HDL-C	2.28 1.51–2.73	3.32 2.75–3.63	3.30 2.71–4.53	Row factor	F (19,26) = 1.162	0.355
				Column factor	F (2,26) = 9.078	0.001 *
AIP (log10 TG/HDL)	0.36 0.23–0.44	0.52 0.44–0.56	0.53 0.43–0.66	Row factor	F (19,26) = 1.583	0.137
				Column factor	F (2,26) = 10.290	0.001 *
LAP	45.69 37.31–51.78	42.90 37.72–56.45	77.31 62.42–97.09	Row factor	F (19,26) = 1.011	0.481
				Column factor	F (2,26) = 13.330	0.001 *
Irisin (ng/mL)	246.76 219.67–465.92	223.97 212.26–393.03	219.45 205.09–257.25	Row factor	F (19,26) = 1.176	0.345
				Column factor	F (2,26) = 0.679	0.516
	***T2DM***					
	**NTEW** **(*n* = 40)**	**ETNW** **(*n* = 21)**	**HTGW** **(*n* = 29)**	**Factor**	**F** **(DFn, DFd)**	***p*** **value**
Insulin (µU/mL)	49.73 25.60–51.53	48.19 43.89–51.03	50.95 46.70–52.51	Row factor	F (39,48) = 2.284	0.003 *
				Column factor	F (2,48) = 0.404	0.670
HOMA-IR	17.79 11.24–21.68	17.91 15.81–22.52	18.69 17.01–21.66	Row factor	F (39,48) = 6.588	<0.0001 *
				Column factor	F (2,48) = 0.422	0.658
QUICKI	0.11 0.11–0.12	0.11 0.11–0.12	0.11 0.11–0.12	Row factor	F (39,48) = 5.452	<0.0001 *
				Column factor	F (2,48) = 0.128	0.881
TG/HDL-C	3.06 2.32–3.78	6.23 4.19–9.03	4.44 3.55–5.90	Row factor	F (39,48) = 0.539	0.976
				Column factor	F (2,48) = 17.100	<0.0001 *
AIP (log10 TG/HDL)	0.49 0.36–0.58	0.79 0.62–0.96	0.65 0.55–0.77	Row factor	F (39,48) = 0.678	0.898
				Column factor	F (2,48) = 22.940	<0.0001 *
LAP	78.58 53.11–88.03	65.71 47.23–124.80	112.00 96.84–152.90	Row factor	F (39,48) = 0.827	0.728
				Column factor	F (2,48) = 18.580	<0.0001 *
Irisin (ng/mL)	141.09 112.61–222.16	235.64 144.02–246.45	158.33 138.12–217.84	Row factor	F (39,48) = 0.662	0.907
				Column factor	F (2,48) = 4.857	0.012 *

HOMA-IR: Homeostatic Model Assessment of Insulin Resistance; QUICKI: Quantitative Insulin Sensitivity Check Index; AIP: atherogenic index of plasma; LAP: lipid accumulation product; *: *p* < 0.05: statistically significant.

**Table 4:** Post hoc pairwise comparisons among hypertriglyceridemic-waist (HTGW) phenotypes (Dunn test with Holm–Bonferroni correction) in prediabetes (PreDM) and type 2 diabetes (T2DM).

Group	Variable	Comparison	Kruskal–Wallis *p*	Mann–Whitney U	*p* (Raw)	*p* (Holm-Adjusted)
PreDM	Insulin	NTEW vs. ETNW	0.994	133	0.927	1.000
	Insulin	NTEW vs. HTGW	0.994	146	0.907	1.000
	Insulin	ETNW vs. HTGW	0.994	97	1.000	1.000
	HOMA-IR	NTEW vs. ETNW	0.559	155	0.367	1.000
	HOMA-IR	NTEW vs. HTGW	0.559	151	0.987	1.000
	HOMA-IR	ETNW vs. HTGW	0.559	76	0.333	1.000
	QUICKI	NTEW vs. ETNW	0.559	105	0.367	1.000
	QUICKI	NTEW vs. HTGW	0.559	149	0.987	1.000
	QUICKI	ETNW vs. HTGW	0.559	119	0.333	1.000
	TG/HDL-C	NTEW vs. ETNW	<0.0001	40	0.001	0.002 *
	TG/HDL-C	NTEW vs. HTGW	<0.0001	46	0.001	0.002 *
	TG/HDL-C	ETNW vs. HTGW	<0.0001	91	0.782	0.782
	AIP	NTEW vs. ETNW	<0.0001	40	0.001	0.002 *
	AIP	NTEW vs. HTGW	<0.0001	46	0.001	0.002 *
	AIP	ETNW vs. HTGW	<0.0001	91	0.782	0.782
	LAP	NTEW vs. ETNW	<0.0001	130	1.000	1.000
	LAP	NTEW vs. HTGW	<0.0001	46	0.001	0.002 *
	LAP	ETNW vs. HTGW	<0.0001	25	0.001	0.002 *
	Irisin	NTEW vs. ETNW	0.374	160	0.277	0.710
	Irisin	NTEW vs. HTGW	0.374	186	0.237	0.710
	Irisin	ETNW vs. HTGW	0.374	106	0.713	0.713
T2DM	Insulin	NTEW vs. ETNW	0.104	410	0.885	0.885
	Insulin	NTEW vs. HTGW	0.104	420	0.053	0.158
	Insulin	ETNW vs. HTGW	0.104	218	0.091	0.182
	HOMA-IR	NTEW vs. ETNW	0.387	368	0.434	0.869
	HOMA-IR	NTEW vs. HTGW	0.387	469	0.179	0.538
	HOMA-IR	ETNW vs. HTGW	0.387	286	0.724	0.869
	QUICKI	NTEW vs. ETNW	0.387	472	0.434	0.869
	QUICKI	NTEW vs. HTGW	0.387	691	0.179	0.538
	QUICKI	ETNW vs. HTGW	0.387	323	0.724	0.869
	TG/HDL-C	NTEW vs. ETNW	<0.0001	44	<0.0001	<0.0001 *
	TG/HDL-C	NTEW vs. HTGW	<0.0001	223	<0.0001	<0.0001 *
	TG/HDL-C	ETNW vs. HTGW	<0.0001	442	0.007	0.007
	AIP	NTEW vs. ETNW	<0.0001	44	<0.0001	<0.0001 *
	AIP	NTEW vs. HTGW	<0.0001	223	<0.0001	<0.0001 *
	AIP	ETNW vs. HTGW	<0.0001	442	0.007	0.007 *
	LAP	NTEW vs. ETNW	<0.0001	436	0.814	0.814
	LAP	NTEW vs. HTGW	<0.0001	85	<0.0001	<0.0001 *
	LAP	ETNW vs. HTGW	<0.0001	129	0.001	0.001 *
	Irisin	NTEW vs. ETNW	0.005	228.5	0.004	0.011 *
	Irisin	NTEW vs. HTGW	0.005	477	0.213	0.213
	Irisin	ETNW vs. HTGW	0.005	438	0.009	0.018 *

HOMA-IR: Homeostatic Model Assessment of Insulin Resistance; QUICKI: Quantitative Insulin Sensitivity Check Index; AIP: atherogenic index of plasma; LAP: lipid accumulation product; *: *p* < 0.05: statistically significant.

**Table 5:** Multiple linear regression (MLR) models evaluating independent associations between circulating irisin levels and metabolic parameters in prediabetic (PreDM) participants.

PreDM					
**Model 1 (AIP) | n = 48 | R^2^ = 0.124, adj. R^2^ = −0.083, Model *p* = 0.789—Coefficients**					
**Variable**	**Beta**	**SE**	**t**	***p***	**VIF**
Intercept	806.875	716.267	1.126	0.267	
AIP (log10 TG/HDL)	−150.987	238.753	−0.632	0.531	8.01
Insulin (µU/mL)	24.323	50.251	0.484	0.631	25.21
HbA1c (%)	−135.958	85.050	−1.599	0.118	1.13
HOMA-IR	−26.381	181.110	−0.146	0.885	27.7
QUICKI	1720.462	2479.892	0.694	0.492	5.91
TG/HDL-C	21.488	42.635	0.504	0.617	8.19
Waist (cm)	0.306	2.119	0.144	0.886	1.39
Age (y)	−1.822	1.797	−1.014	0.317	1.45
Sex (Female)	−1.014	38.085	−0.027	0.979	1.19
**Model 2 (LAP) | n = 48 | R^2^ = 0.121, adj.R^2^ = −0.087, model *p* = 0.805—Coefficients**					
**Variable**	**Beta**	**SE**	**t**	***p***	**VIF**
Intercept	739.7937	713.6633	1.037	0.306	
LAP	0.6015	1.227	0.49	0.627	3.5
Insulin (µU/mL)	26.1784	50.7105	0.516	0.609	25.56
HbA1c (%)	−126.966	84.6274	−1.5	0.142	1.11
HOMA-IR	−18.7014	181.1387	−0.103	0.918	27.6
QUICKI	2176.018	2577.444	0.844	0.404	6.36
TG/HDL-C	−11.9172	24.8536	−0.479	0.634	2.77
Age (y)	−2.0139	1.7893	−1.126	0.267	1.43
Sex (Female)	−11.5772	44.759	−0.259	0.797	1.64

Two separate models were tested: Model 1 included the atherogenic index of plasma (AIP). In contrast, Model 2 included the lipid accumulation product (LAP), with all variables adjusted for insulin, HbA1c, HOMA-IR, QUICKI, TG/HDL-C, waist circumference, age, and sex.

**Table 6:** Multiple linear regression (MLR) models evaluating independent associations between circulating irisin levels and metabolic parameters in patients with type 2 diabetes mellitus (T2DM).

T2DM					
**Model 1 (AIP) | n = 90 | R^2^ = 0.096, adj. R^2^ = −0.006, Model *p* = 0.494—Coefficients**					
**Variable**	**Beta**	**SE**	**t**	***p***	**VIF**
Intercept	530.0145	792.9969	0.668	0.506	
AIP (log10 TG/HDL)	−47.3986	120.8161	−0.392	0.696	8.84
Insulin (µU/mL)	−1.6927	2.2641	−0.748	0.457	8.97
HbA1c (%)	−1.0703	5.7341	−0.187	0.852	1.69
HOMA-IR	0.7334	4.3827	0.167	0.868	8.49
QUICKI	−487.184	5995.548	−0.081	0.935	17.14
TG/HDL-C	1.0372	9.3218	0.111	0.912	8.77
Waist (cm)	−1.708	0.7065	−2.418	0.018	1.36
Age (y)	−0.1053	0.8743	−0.12	0.904	1.12
Sex (Female)	−5.5029	18.1258	−0.304	0.762	1.06
**Model 2 (LAP) | n = 90 | R^2^ = 0.126, adj.R^2^ = 0.028, model *p* = 0.261—Coefficients**					
**Variable**	**Beta**	**SE**	**t**	***p***	**VIF**
Intercept	706.4649	785.049	0.9	0.371	
LAP	0.5772	0.3382	1.706	0.092	3.5
Insulin (µU/mL)	−2.4167	2.2	−1.098	0.275	8.76
HbA1c (%)	−1.5802	5.5334	−0.286	0.776	1.62
HOMA-IR	0.5701	4.3061	0.132	0.895	8.48
QUICKI	−851.594	5889.391	−0.145	0.885	17.1
TG/HDL-C	−9.6776	5.4741	−1.768	0.081	3.13
Age (y)	0.1668	0.8718	0.191	0.849	1.15
Sex (Female)	−17.006	19.1175	−0.89	0.376	1.22

Two separate models were tested: Model 1 included the atherogenic index of plasma (AIP), while Model 2 included the lipid accumulation product (LAP), both adjusted for insulin, HbA1c, HOMA-IR, QUICKI, TG/HDL-C, waist circumference, age, and sex.

**Table 7:** Parsimonious multiple linear regression models (AIP, LAP) in the PreDM cohort.

PreDM					
**Model 1 (AIP): Standardized Coefficients | n = 48 | R^2^ = 0.104, Adj. R^2^ = −0.003, Model *p* = 0.444**					
**Predictor**	**βstd**	**SE**	**t**	***p***	**VIF**
Intercept	0	0.146	0	1	
AIP (log10 TG/HDL)	−0.083	0.165	−0.503	0.617	1.280
HbA1c (%)	−0.237	0.150	−1.584	0.121	1.050
Age (y)	−0.160	0.164	−0.972	0.336	1.270
Sex (Female)	−0.006	0.156	−0.036	0.972	1.140
Waist (cm)	0.029	0.159	0.180	0.858	1.190
**Model 2 (LAP): Standardized coefficients | n = 48 | R^2^ = 0.098, Adj. R^2^ = 0.010, model *p* = 0.335**					
**Predictor**	**βstd**	**SE**	**t**	***p***	**VIF**
Intercept	0.0001	0.143	0.0001	1.000	
LAP	−0.172	0.160	−1.074	0.288	1.380
HbA1c (%)	−0.218	0.147	−1.486	0.144	1.120
Age (y)	−0.151	0.161	−0.939	0.353	1.090
Sex (Female)	0.033	0.152	0.216	0.830	1.040

**Table 8:** Parsimonious multiple linear regression models (AIP, LAP) in the T2DM cohort.

T2DM					
**Model 1 (AIP): Standardized Coefficients | n = 90 | R^2^ = 0.131, Adj. R^2^ = 0.027, Model *p* = 0.271**					
**Predictor**	**βstd**	**SE**	**t**	***p***	**VIF**
Intercept	0.0001	0.109	0.0001	1.000	
AIP (log10 TG/HDL)	−0.118	0.118	−0.999	0.321	1.44
HbA1c (%)	−0.106	0.108	−0.980	0.330	1.160
Age (y)	−0.098	0.111	−0.883	0.380	1.110
Sex (Female = 1)	0.049	0.106	0.462	0.645	1.050
Waist (cm)	0.086	0.109	0.789	0.433	1.330
**Model 2 (LAP): Standardized coefficients | n = 90 | R^2^ = 0.154, Adj. R^2^ = 0.081, model *p* = 0.087**					
**Predictor**	**βstd**	**SE**	**t**	***p***	**VIF**
Intercept	0.0001	0.101	0.0001	1.000	
LAP	−0.204	0.105	−1.942	0.056	1.390
HbA1c (%)	−0.112	0.098	−1.144	0.256	1.110
Age (y)	−0.097	0.100	−0.972	0.334	1.100
Sex (Female = 1)	0.041	0.095	0.431	0.668	1.040

**Table 9:** Logistic regression for the hypertriglyceridemic-waist (HTGW) phenotype (adjusted for age (y), sex (Male = 1), BMI (kg/m^2^), group (T2DM = 1), HbA1c (%), and insulin (µU/mL) as predictors).

Variable	OR	95% CI	*p*-Value
Intercept	54.01	0.54–5357.62	0.089
Irisin_z (per 1 SD)	0.94	0.59–1.52	0.814
Age (y)	0.94	0.90–0.99	0.015
Sex (Male = 1)	0.12	0.04–0.36	<0.001
BMI (kg/m^2^)	1.00	0.94–1.07	0.956
Group (T2DM = 1)	0.27	0.02–3.80	0.329
HbA1c (%)	1.42	1.00–2.00	0.048
Insulin (µU/mL)	1.00	0.95–1.05	0.964

Model statistics: LR χ^2^ (7) = 33.42, *p* < 0.001 (global significant); Nagelkerke R^2^ = 0.317; Hosmer–Lemeshow χ^2^ (8) = 10.57; *p* = 0.227 (good calibration). χ^2^—likelihood ratio Chi-square statistic test for overall model significance; Nagelkerke R^2^—proportion of variance in HTGW status explained by the model; Hosmer–Lemeshow—test of calibration (*p* > 0.05 indicates good fit).

**Table 10:** Logistic regression for the hypertriglyceridemic-waist (HTGW) phenotype (adjusted for age (y), sex (Male = 1), BMI (kg/m^2^), group (T2DM = 1), HbA1c (%), and insulin (µU/mL) as predictors) in PreDM individuals.

Variable	OR	95% CI	*p*
Intercept	2.88	0.02–478.6	0.681
Irisin-z (per 1 SD)	0.72	0.35–1.47	0.362
Age (y)	0.95	0.89–1.02	0.141
Sex (Male = 1)	0.21	0.05–0.81	0.024
BMI (kg/m^2^)	1.01	0.91–1.11	0.913
HbA1c (%)	1.30	0.84–2.02	0.241
Insulin (µU/mL)	1.04	0.97–1.12	0.248

*Model fit*: χ^2^(6) = 15.87, *p* = 0.014; Nagelkerke R^2^ = 0.297; Hosmer–Lemeshow χ^2^(8) = 6.42, *p* = 0.602; AUC = 0.794, 95% CI: 0.674–0.883.

**Table 11:** Logistic regression for the hypertriglyceridemic-waist (HTGW) phenotype (adjusted for age (y), sex (Male = 1), BMI (kg/m^2^), group (T2DM = 1), HbA1c (%), and insulin (µU/mL) as predictors) in T2DM individuals.

Variable	OR	95% CI	*p*
Intercept	12.42	0.25–603.4	0.210
Irisin-z (per 1 SD)	1.08	0.57–2.01	0.810
Age (y)	0.93	0.88–0.99	0.020
Sex (Male = 1)	0.18	0.05–0.63	0.008
BMI (kg/m^2^)	0.99	0.92–1.08	0.924
HbA1c (%)	1.45	1.01–2.09	0.046
Insulin (µU/mL)	1.02	0.96–1.09	0.471

*Model fit*: χ^2^(6) = 19.56, *p* = 0.003; Nagelkerke R^2^ = 0.338; Hosmer–Lemeshow χ^2^(8) = 8.93, *p* = 0.347; AUC = 0.813, 95% CI: 0.725–0.893.

**Table 12:** Correlations between serum irisin and metabolic parameters in prediabetic (PreDM) participants.

Myokine	Variable	rho	*p*-Value	Interpretation
Irisin	Insulin	−0.211	0.094	Negative, non-significant
	HbA1c (%)	−0.178	0.156	Slight inverse trend, NS
	HOMA-IR	−0.228	0.072	Moderate inverse trend, borderline
	QUICKI	0.232	0.068	Positive trend, borderline
	LAP	−0.312	0.010	Significant negative correlation
	AIP	−0.274	0.022	Significant negative correlation
	TG/HDL-C	−0.268	0.025	Significant negative correlation
	HbA1c	−0.184	0.142	Negative, non-significant

**Table 13:** Correlations between serum irisin and metabolic parameters in type 2 diabetes mellitus (T2DM) participants.

Myokine	Variable	rho	*p*-Value	Interpretation
Irisin	Insulin	−0.316	0.008	Significant negative correlation
	HbA1c (%)	−0.241	0.041	Weak inverse correlation
	HOMA-IR	−0.384	<0.001	Strong negative correlation
	QUICKI	0.382	<0.001	Strong positive correlation
	LAP	−0.268	0.002	Moderate negative correlation
	AIP	−0.240	0.005	Significant inverse correlation
	TG/HDL-C	−0.240	0.005	Significant inverse correlation
	Waist	−0.203	0.068	Weak negative, borderline

**Table 14:** Spearman correlations using the Benjamini–Hochberg false discovery rate (FDR) correction in prediabetic (PreDM) and type 2 diabetes (T2DM) participants.

Group	Variable	N	*ρ*	*p* (Raw)	*p* (FDR)
Prediabetes	Insulin (µU/mL)	48	−0.086	0.562	0.643
	HbA1c (%)	48	−0.319	0.027	0.217
	HOMA-IR	48	−0.144	0.329	0.449
	QUICKI	48	0.124	0.309	0.409
	LAP	48	−0.022	0.88	0.880
	AIP (log10 TG/HDL)	48	−0.142	0.336	0.349
	TG/HDL-C	48	−0.132	0.326	0.429
	Waist (cm)	48	0.205	0.163	0.249
T2DM	Insulin (µU/mL)	90	−0.261	0.013	0.104
	HbA1c (%)	90	0.082	0.440	0.931
	HOMA-IR	90	−0.009	0.931	0.951
	QUICKI	90	0.029	0.931	0.831
	LAP	90	−0.022	0.841	0.661
	AIP (log10 TG/HDL)	90	−0.038	0.525	0.531
	TG/HDL-C	90	−0.059	0.445	0.441
	Waist (cm)	90	−0.077	0.722	0.451

**Table 15:** Formulas utilized in the evaluation of insulin resistance and the metabolic profile.

Index	Formula
HOMA-IR	(Insulin [µU/mL] × Glucose [mg/dL])/405 [73]
QUICKI	1/(log (Insulin [µU/mL]) + log (Glucose [mg/dL])) [74]
AIP	log10 (TG/HDL-C) [75]
LAP	males = (WC [cm] − 65) × TG [mmol/L]; females = (WC [cm] − 58) × TG [mmol/L] [76]

## Data Availability

The data used to support the findings of this study are available from the corresponding author upon reasonable request.
